# Non-oxidized porous silicon-based power AC switch peripheries

**DOI:** 10.1186/1556-276X-7-566

**Published:** 2012-10-11

**Authors:** Samuel Menard, Angélique Fèvre, Damien Valente, Jérôme Billoué, Gaël Gautier

**Affiliations:** 1 Université François Rabelais de Tours, GREMAN UMR CNRS 7347, 16 Rue Pierre et Marie Curie, BP 7155, Tours Cedex 2, 37071, France; 2 ST Microelectronics, 16 Rue Pierre et Marie Curie, Tours Cedex 2, 37071, France

**Keywords:** Anodic etching, Meso- and microporous silicon, AC switch periphery, Electrical characterization

## Abstract

We present in this paper a novel application of porous silicon (PS) for low-power alternating current (AC) switches such as triode alternating current devices (TRIACs) frequently used to control small appliances (fridge, vacuum cleaner, washing machine, coffee makers, etc.). More precisely, it seems possible to benefit from the PS electrical insulation properties to ensure the OFF state of the device. Based on the technological aspects of the most commonly used AC switch peripheries physically responsible of the TRIAC blocking performances (leakage current and breakdown voltage), we suggest to isolate upper and lower junctions through the addition of a PS layer anodically etched from existing AC switch diffusion profiles. Then, we comment the voltage capability of practical samples emanating from the proposed architecture. Thanks to the characterization results of simple Al-PS-Si(P) structures, the experimental observations are interpreted, thus opening new outlooks in the field of AC switch peripheries.

## Background

Up to now, porous silicon is widely investigated for sensing, photonic, or MEMS applications as it is well summarized in [1], but its mesoporous or microporous electrical properties are not massively exploited. Two main topics have been discussed in the literature. First, the integration of inductances on the top of micro- or mesoporous layers improves their quality factor by lowering Eddy currents in the substrate [2]. Second, the isolation of silicon islands, where bipolar or MOS transistors may be integrated, has been studied in [3-5] for the development of a novel integrated circuit technology. However, most of the time, porous silicon is oxidized in order to improve its dielectric performances.

Nevertheless, several research teams have demonstrated that depending on bulk properties and anodic etching conditions, porous silicon behaves like an insulator. More precisely, its porosity is higher and its dielectric constant and conductivity are lower as illustrated in [6,7]. It should be noted too that high porosities generate a thermal activation increase of the porous silicon conductivity.

High porosities (>50%) may be reached through p-type substrates with doping levels between 10^16^ and 10^18^/cm^3^ as explained in [8]. Then, knowing that such p-type layers may be easily found in power alternating-current (AC) switch technologies, and knowing that insulation is one of the major issues of this kind of structures, it is interesting to study how porous silicon may bring improvements for such devices.

The first part of the present paper will remind the most important features to know about AC switches and their associated technologies. We will also give the first schematic of the novel AC switch architecture that we would like to target. Then, we will describe our experimental structure, on which electrical assessments have been performed. Finally, the results will be discussed on the basis of previous observations done on simple vertical metal-porous silicon-silicon (metal-PS-Si(P)) structures.

## Methods

### AC switch peripheries: state of the art

**Figure 1:**
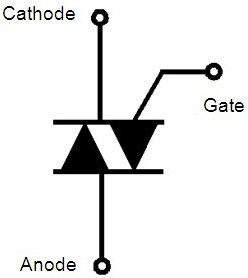
**Electrical symbol of the TRIAC.** The anode and cathode electrodes are the power terminals, while the gate is the command pin.

**Figure 2:**
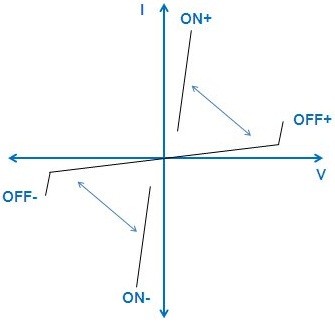
***I*-*V *characteristic of the TRIAC.** The low and high impedance states are denoted as ON and OFF, respectively. If the anode of the TRIAC is positively biased against the cathode, the ‘+’ sign is used; else, the ‘−’ sign is adopted. The bidirectional arrows indicate the switching from one state to the other in accordance to the signal applied to the gate.

AC switches are generally used to control low-power loads connected to the mains for home appliance applications such as fridge compressors, washing machine drums, coffee machine resistances, and so on. Most of AC switches are triode alternating current devices (TRIACs), whose electrical symbol and characteristics are presented in Figures 1 and 2, respectively. The anode and cathode terminals manage the power part of the TRIAC. Depending on the desired working of the load, the TRIAC has to switch from the OFF state, where it withstands high voltages with leakage currents as low as possible, to the ON state with a minimum of conduction losses. The switching behavior of the TRIAC is another important feature to take care at the application level, which is controlled by the gate terminal. Because of the direct connection to the mains, the TRIAC is bidirectional in voltage and current [9,10].

**Figure 3:**
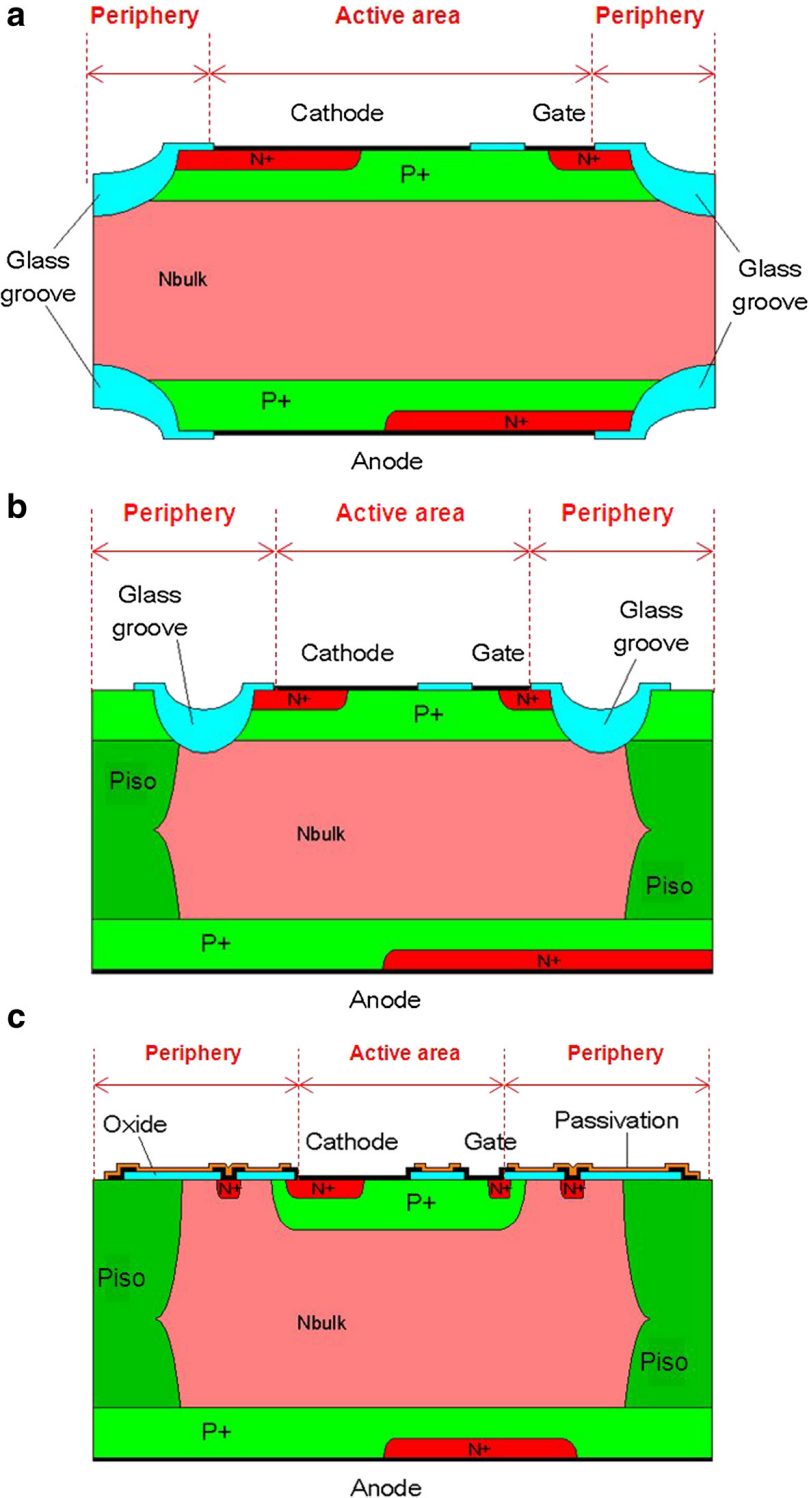
**Schematic cross section of AC switch technologies: (a) *double mesa*, (b) *top glass*, (c) *planar*.** The anode, cathode, and gate terminals are indicated for each case. The peripheries and active areas are delimited through the red dotted lines.

All power AC switch devices are manufactured through three main technologies: *double mesa*, *top glass*, and *planar* (Figure 3a,b,c, respectively). Whatever the technology is, the TRIAC architecture remains unchanged. The active area is located in the center of the die, and the periphery encompasses all around. The active area is responsible of the ON performances, while the periphery controls the OFF ones. Note that the switching behavior is the result of both the periphery and active area designs.

Usually, the name of the technology corresponds to the way the periphery is achieved. In the case of the *double mesa*, grooves are etched from the n-type silicon substrate (N_bulk_) on both sides of the die. They are then filled with glass, the most appropriate material when thick passivation layers are needed. For the *top glass*, deep p-wells are diffused from both sides of the wafer and joined to form through wafer p-wells (P_iso_). The insulation is then ensured by top side grooves filled with glass. The structure of the *planar* periphery is based on the *top glass* one, except that the p+ doping of the active area is localized through a standard photolithography step, thus allowing the use of thinner passivation layers.

**Figure 4:**
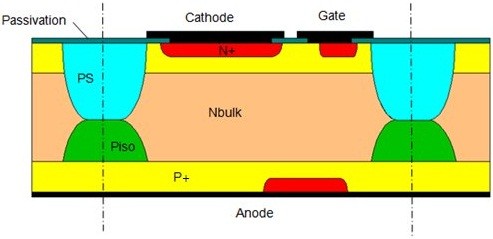
**TRIAC periphery using porous silicon as a junction termination.** ‘PS’ and ‘P_iso_’ mean porous silicon and through wafer p-well, respectively.

Each of these technologies has advantages and drawbacks. According to the application needs, the best device will result from the trade-off between the blocking capability, the conduction losses, the commutation performances, and the reliability. The cost attractiveness is an important element of comparison too. As the ideal technology does not exist yet, porous silicon, thanks to its electrical properties and its easiness to manufacture, is believed to offer a new path in the field of power AC switch technologies, that is to say TRIAC peripheries. More precisely, we expect to target powerful peripheries with a gain of space (i.e., cost saving) by isolating both faces of the die with a porous silicon layer etched from the through wafer p-wells commonly encountered in *top glass* or *planar* technologies. A possible solution is sketched in Figure 4.

### Experimental section

**Figure 5:**
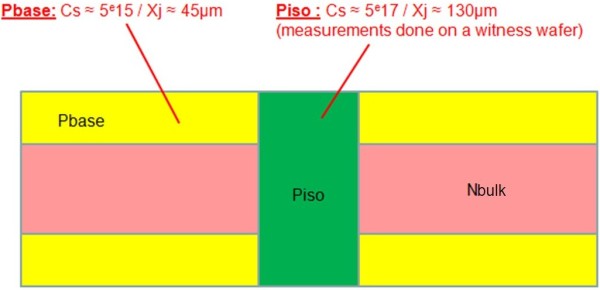
**Schematic cross section of the experimental wafer before anodization.** The p-type P_iso_ and P_base_ layers are formed in a 33- to 39-Ω cm n-type substrate (N_bulk_). The junction depth *X*_j_ and the surface concentration *C*_s_ are given for both diffusions.

First, through wafer p-wells (P_iso_) were formed on a 33- to 39-Ω cm n-type substrate. Then, a moderately doped p-type diffusion (P_base_) was implemented on the whole surfaces of the wafer (top and bottom). The resulting structure, described in Figure 5, corresponds to most of AC switch initial process stages.

Then, anodic etching was carried out on top of the wafer in a 6-in. double-tank anodization cell filled with a 30% HF-H_2_O-acetic acid electrolyte, applying a constant current density of 30 mA/cm^2^ for 60 min. Even if the cell allows 6-in. treatments, the anodization here was restricted to a 1-in. circular surface. After etching, the wafer was dried at 150°C for a few minutes in an ambient atmosphere.

**Figure 6:**
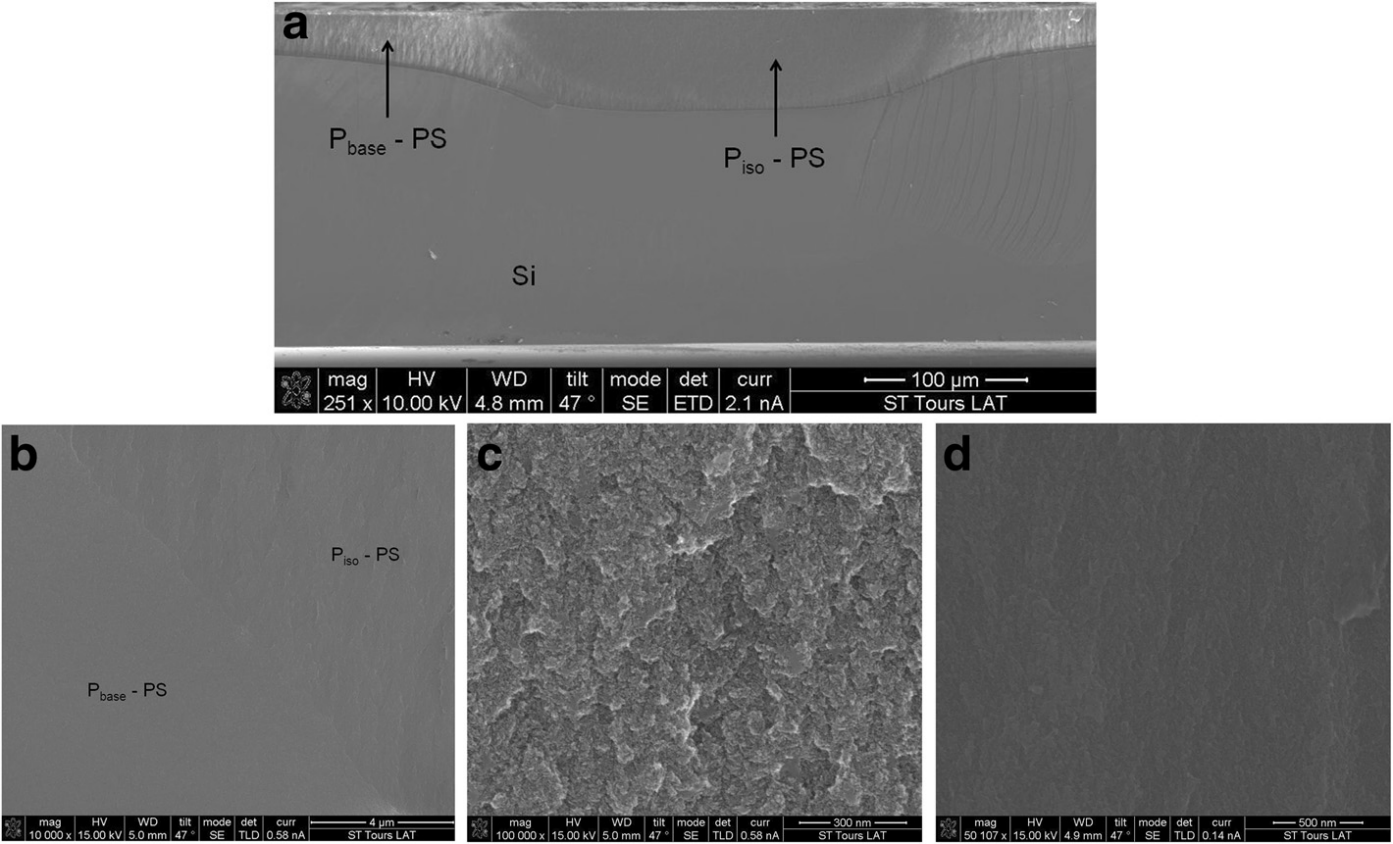
**Cross-sectional SEM images of porous silicon layers formed after the anodization of the experimental wafer:** (**a**) overall view, P_iso_-PS and P_base_-PS indicate the porous silicon layers formed from the P_iso_ and P_base_ diffusions, respectively; (**b**) focus on the frontier between P_iso_-PS and Pbase-PS areas; (**c**) morphology of the P_iso_-PS layer; (**d**) morphology of the P_base_-PS layer.

**Figure 7:**
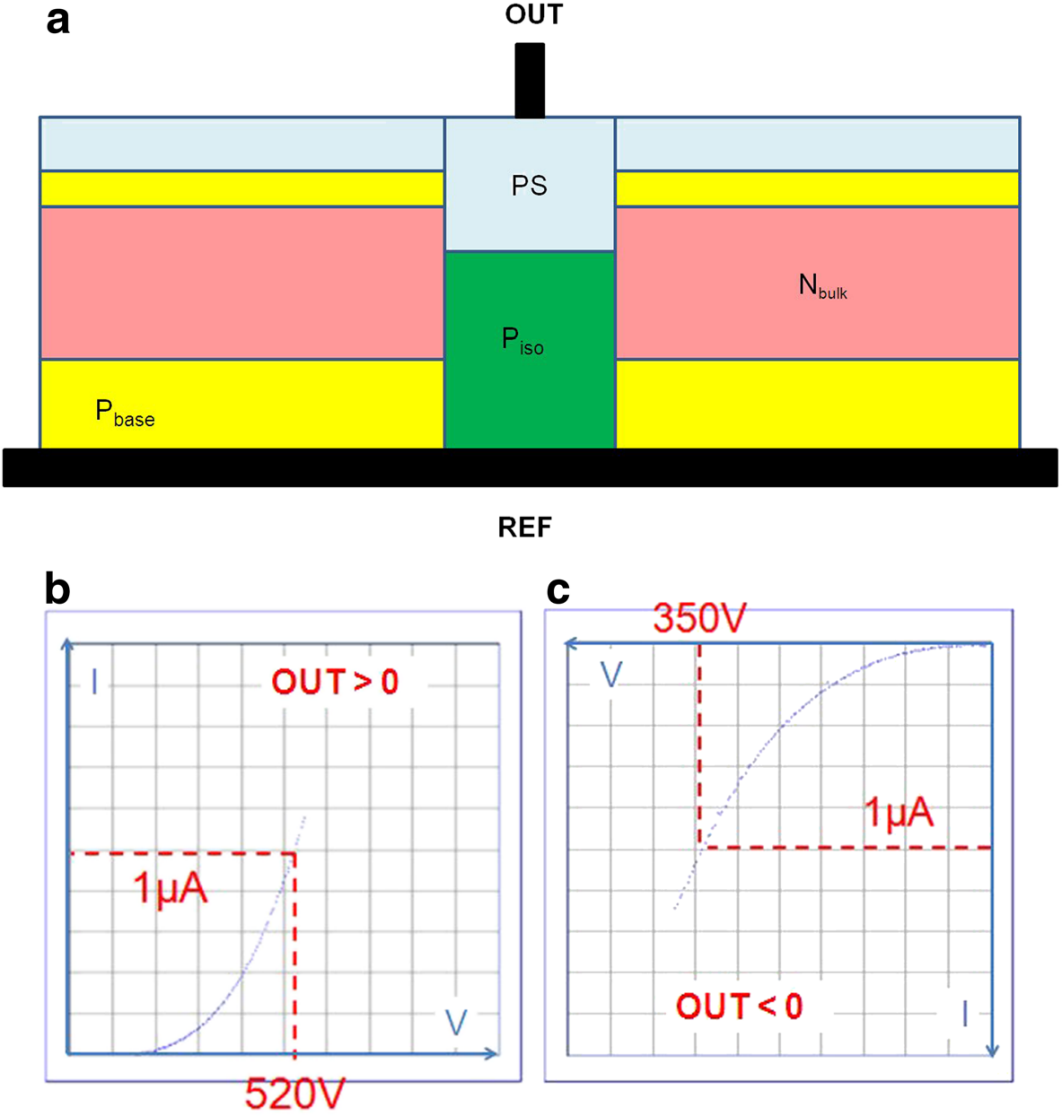
**Electrical performance assessment of the porous silicon-based experimental structure:** (**a**) measurement setup, (**b**) forward bias results (100 V/division on the voltage axis and 200 nA/division on the current axis), (**c**) reverse bias results (50 V/division on the voltage axis and 200 nA/division on the current axis). REF and OUT are the reference and power terminals, respectively.

The above experimental flow leads to porous silicon layer thicknesses of 60 μm (P_iso_-PS) and 20 μm (P_base_-PS), respectively, from P_iso_ and P_base_ diffusions. The measurements were performed by scanning electron microscopy (SEM) on sample cross sections as presented on Figure 6a. The physical properties of the porous silicon layer depend on the doping profile from which it is formed [11]. It is then not surprising to observe different porous silicon aspects on Figure 6a and, more precisely, on Figure 6b where the focus was done at the frontier of both P_iso_-PS and P_base_-PS areas. Based on [11], we may expect the formation of mesoporous and microporous layers, respectively, from the P_iso_ and P_base_ diffusions even if in our case, the doping profiles are not homogeneous, as usually reported, but gradual. By further increasing the magnification, the P_iso_-PS porous silicon layer (Figure 6c) reveals a sponge-like morphology without any preferential pore orientation. The pore diameter is difficult to assess because of cleavage effects, but it was estimated between 5 and 10 nm, thus confirming the expected mesoporous nanostructure. This typical morphology is coherent with observations presented in [11,12]. The P_base_-PS structure is more difficult to observe. The sponge-like aspect is always visible as confirmed on Figure 6d, but this time, the pore size could not be evaluated because of their small size, probably lower than 2 nm. A more precise TEM view was given in [13]. From these observations, it is clear that the resulting porous silicon layers involve the isolation of the P_base_/N_bulk_ junction as schematically drawn on Figure 7a.

The breakdown capability of the porous silicon layer obtained from the samples described previously was assessed on a probe station connected to a Tektronix 370A curve tracer (Tektronix Ltd., Bracknell, UK). A metallic chuck is used in order to connect the back side of the wafer, and one probe is positioned on top. Note that no metal layer was used to facilitate the electrical contact as it was not supposed to impact the results as discussed in the ‘Results and discussion’ section. All measurements were carried out at ambient temperature and in the dark. Figure 7 depicts the experimental setup and typical electrical results in both forward and reverse biases. At a current level of 1 μA, a usual reference value for AC switches, the porous silicon structure demonstrates voltage capabilities higher than 520 and 350 V with positive and negative biases, respectively. In parallel, some samples coming from the same experimentation but without any porous silicon layers were tested with voltage results lower than 30 V at 1 μA. The insulation character of the porous silicon layer is then confirmed.

## Results and discussion

Despite the simplicity of the structure (large surface pattern, no PS localization, no specific treatment of the porous silicon layer, no metal contact, no passivation layers), interesting voltage blocking capabilities have been reached without any sample destruction and with good reproducibility. Another interesting point is the non-symmetric behavior between forward and reverse biases.

To interpret the results, we need to clarify previous results obtained on simple metal-PS-Si(P) structures. Table 1 presents a synthesis of the works of different research teams. Depending on the substrate resistivities, the PS anodization conditions, and the nature of the metal layer used for the electrical contacts on PS, current–voltage (*I*-*V*) characteristics are ohmic or nonlinear.

**Table 1:** **Process conditions and electrical *I*-*V *results found in different electrical studies of vertical metal-PS-Si(P) structures**

**Paper reference**	**P-type substrate**		**C**_**El**_	***J***_**etch**_**(mA/cm**^**2**^**)**	***P *(%)**	***d***_**PS**_**(nm)**	***t***_**PS**_**(μm)**	**ML**	**PS contact (according to the authors)**	***I*-*V *results**		**Model**
	**O**_**S**_	***ρ***_**S**_**(Ω cm)**								**Range (V)**	**WF**	
Ben-Chorin et al. [14]	100	5	HF (49%) + ethanol (1:1)	30	?	3 to 5	0.5 to 15	Au NiCr In	Quasi-ohmic	[−100, +100]	Quasi-linear^a^	BC
Ben-Chorin et al. [14]	100	0.075	HF (49%) + ethanol (1:1)	100	?	3 to 5	0.5 to 15	Au NiCr In	Quasi-ohmic	[−100, +100]	Quasi-linear^a^	BC
Balagurov et al. [15]	111	0.005	HF (48%) + ethanol (5:1)	13	30	>10	1.5 to 30	Au Al	Ohmic	[0, +100]	Linear^b^	AN
Bouaïcha et al. [7]	100	1	HF (40%) + ethanol (1:1)	?	48	2 to 5	?	Al	Ohmic	[0, +5]	Nonlinear	BC
					62							
					75							
Remaki et al. [16]	100	0.02	HF (50%) + ethanol (2:1)	75	50	MESO	1 to 10	Au	Schottky^c^	[−0.5, +0.5]	Quasi-linear^d^	BC
Anderson et al. [17]	100	30 to 40	HF (45%)	10	36	?	0.5 to 5	Al	Schottky	[−0.5, +0.5]	Nonlinear	BC
Anderson et al. [17]	111	0.01 to 0.03	HF (45%)	10	23	?	0.5 to 5	Al	Schottky	[−0.5, +0.5]	Nonlinear	AN
Pulsford et al. [18]	?	25	HF + H_2_O + ethanol (2:3:5)	20	?	?	1.5 and 135	Ca Mg Sb Au Ag	Ohmic^e^	[0, +1]	Nonlinear	BC
Stievenard and Deresmes [19]	100	0.01	HF (40%) + ethanol (1:1)	20	45	MESO	30	Al	Schottky	[−1, +1]	Quasi-linear^d^	AN
Islam et al. [20]	100	6 to 10	HF (48%) + ethanol (1:1)	10	?	MESO	30 to 50	Al	Quasi-ohmic	[−50, +50]	Quasi-linear	BC

All the available information about the starting material (the substrate orientation O_S_ and its resistivity *ρ*_S_), the anodization conditions (the electrolyte composition C_El_ and the etching current density *J*_etch_), the PS features (the porosity *P*, the pore diameter *d*_PS_, and the PS thickness *t*_PS_), the electrical contacts (the metal layer ML used to contact PS and the resulting electrical contact observed according to the authors), and the electrical *IV* results (the studied voltage range and the waveforms WF of the *IV* characteristics) are given. Two models coming from Anderson et al. (AN) [17,19] and Ben-Chorin et al. (BC) [21] are used to interpret all the observations. ^a^Except for PS thickness lower than 2 μm; ^b^space-charge-limited current is observed for PS thicknesses lower than 28 μm; ^c^not true in ambient air (quasi-ohmic); ^d^only in ambient air; ^e^impact of the metal barrier is visible at higher voltage. The question marks were added to avoid bad interpretations of our classification as different behaviors may be observed in the same paper.

We think that all these results may be explained through two models we are going to describe below. We will call the first one ‘Anderson (AN)’ [17] and the second one ‘Ben-Chorin (BC)’ [21].

**Figure 8:**
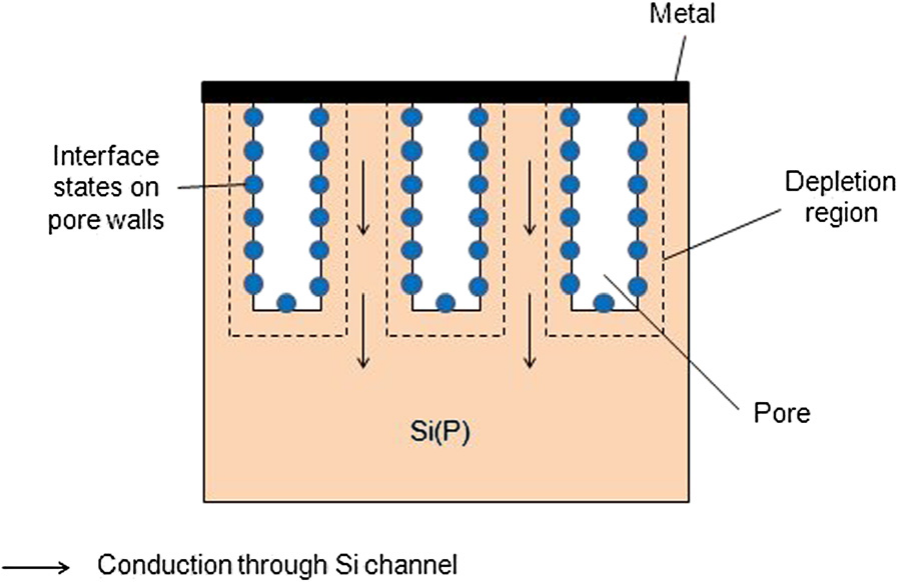
**Anderson model: conduction in columnar porous silicon layers with low porosities from**[17,19].

When the space between neighbored pores is high (about 10 nm and more), depending on the surface state concentration and polarization on pore walls, carriers may flow through Si(P) channels as illustrated on Figure 8[17,19,22]. The channel resistance is modulated by the applied bias, through the extension of the depletion region. According to this model, the metal layer contacts directly the Si(P) substrate, so we should expect an ohmic or rectifying behavior depending on its barrier height. Space-charge-limited current may be observed for higher biases when the depletion regions of adjacent pores meet each other.

This above discussion is easily understandable for columnar pores. If a different pore morphology is achieved, for equivalent PS porosities, we may not observe the same phenomenon. Then, the explanations coming from the BC model for higher PS porosities may be more relevant.

Among all listed studies in Table 1, the results of Balagurov et al. [15], Anderson et al. (low porosity part) [17], and Stievenard and Deresmes [19] seem to be described by the AN model.

**Figure 9:**
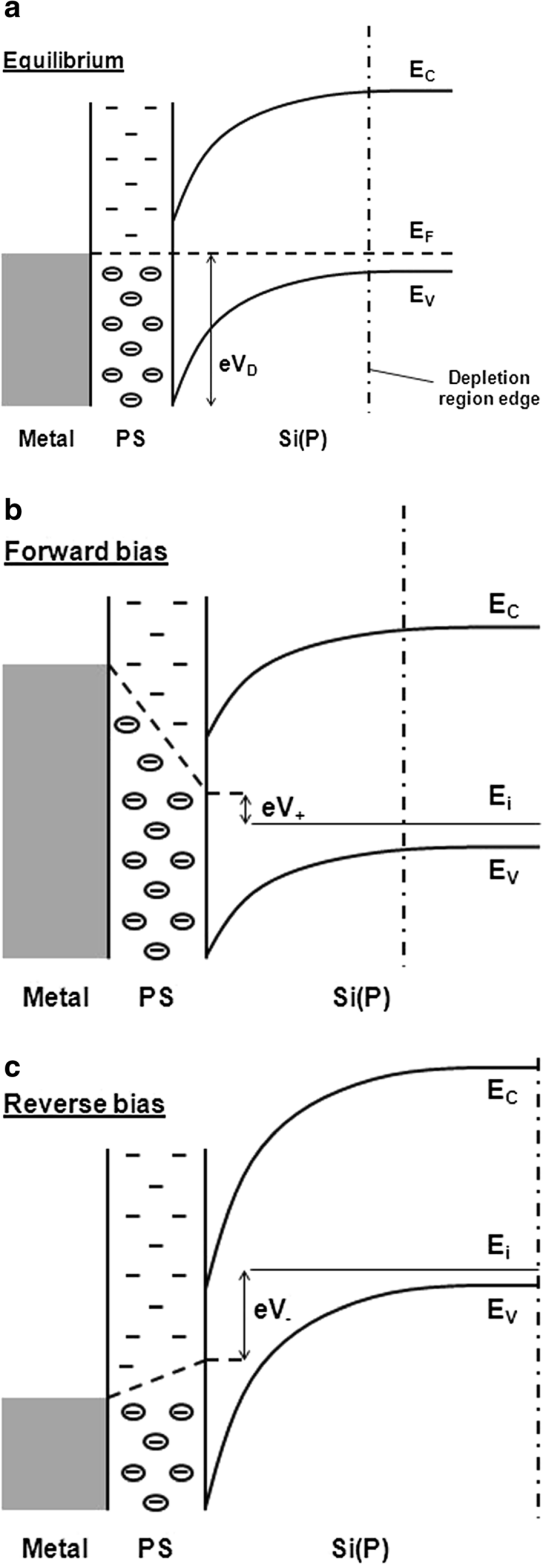
**Schematic band diagram of the structure metal-PS-Si(P) according to Ben-Chorin et al.**[21]**:** (**a**) equilibrium state, (**b**) forward bias (*V*_+_), (**c**) reverse bias (*V*_−_). The Fermi level is represented by the dashed lines. The PS surface states are symbolized by filled circles and single dashes corresponding to occupied and free states, respectively.

When the PS porosity is high, the electrical behavior could be demonstrated by considering the PS/Si(P) interface as a Schottky diode where the metal role is played by the PS layer. Then, the depletion region spreads in the Si(P) substrate. This is possible because of the high density of states on pore walls, which pins the Fermi level at this interface as illustrated by the band diagram of Figure 9a [21]. This representation is only possible if the PS work function is lower than that of the Si(P). The incorporation of H+ ions into the surface of the pores may explain this behavior [23]. Further, it is worth noting that whatever the bias sign and because of the presence of occupied and/or free surface states in the PS layer, a part of the electric field spreads systematically into it, resulting in the PS polarization and the screening of internal fields at the metal/PS interface, that is why the metal/PS contact can be considered as quasi-ohmic. Thus, when the metal-PS-Si(P) structure is forward biased (*V*_+_; Figure 9b), the barrier is decreased from *V*_d_ to *V*_d_ − *V*_+_ and the remaining bias is applied to the PS. Holes are then injected from the Si(P) to the metal, and the PS resistance controls the current flow. If the PS layer thickness is thin (lower than 10 nm), then its resistance is low and the resulting *IV* is rectified. On the other hand, a thick PS layer implies a linear *IV* as the conduction is then dominated by the PS resistance. When the metal-PS-Si(P) structure is reverse biased (*V*_−_; Figure 9c), the barrier is increased from *V*_d_ to *V*_d_ + *V*_−_. The electric field is distributed between the depletion region in the Si(P) and the PS layer. If carriers are generated in the depletion region, the current is then limited by the PS resistance. In both bias cases, the PS resistance is involved in the conduction, that is why a symmetric behavior may be observed. According to Ben-Chorin et al. [24], the conduction in the PS layer is controlled by carrier hopping on the pore wall and/or between all the pores.

Note that Islam et al. [20] proposed another band diagram to describe the band structure of the PS/Si(P) interface. They considered it as a heterojunction. Different working groups have reported such a behavior. For example, based on their photoluminescence measurements, Koshida and Koyama conclude that quantum confinement and/or lattice distortion effects in the PS layer involve a bandgap increase [25]. Even if the BC electrical mechanism does not imply a PS/Si(P) heterojunction, its existence is not questioned, but electrically speaking, the heterojunction may be inactive as the Fermi level is pinned in the energy region of the surface state.

**Figure 10:**
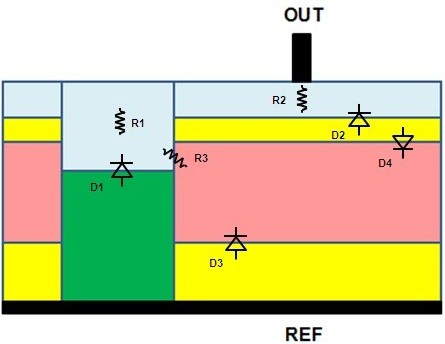
**Equivalent schematic of experimental structure based on association of several diodes (D**_***i***_**) and resistances (R**_***i***_**).**

Based on the previous discussion, we tried to sketch in Figure 10 an equivalent schematic of our experimental structure.

The diode D1 is associated to the PS/P_iso_ interface, where the expected P_iso_ doping should be included between 1 and 100 mΩ cm. Then, the electrical behavior should comply with the BC model as high porosities (>50%) should be reached. The resistance R1 of the PS layer stemming from the P_iso_ anodization will be strongly correlated with D1. Similar to the couple D1/R1, the PS/P_base_ interface may be modeled by the diode D2 associated to its serial PS resistance R2 as the P_base_ doping is expected to be more resistive compared to the P_iso_ one. We have added a PS resistance R3 corresponding to the interface between the n-type substrate and the PS layer. Indeed, as a Schottky-type behavior is observed for the PS/Si(P) interface, thus the PS/Si(N) one should be ohmic. This latter point will be demonstrated in a subsequent paper. Finally, we define D3 and D4 as the reverse and direct P_base_/N_bulk_ junctions, respectively. D3 and D4 typical breakdown voltage is about 1 kV with the involved doping profiles and an appropriate junction termination.

**Figure 11:**
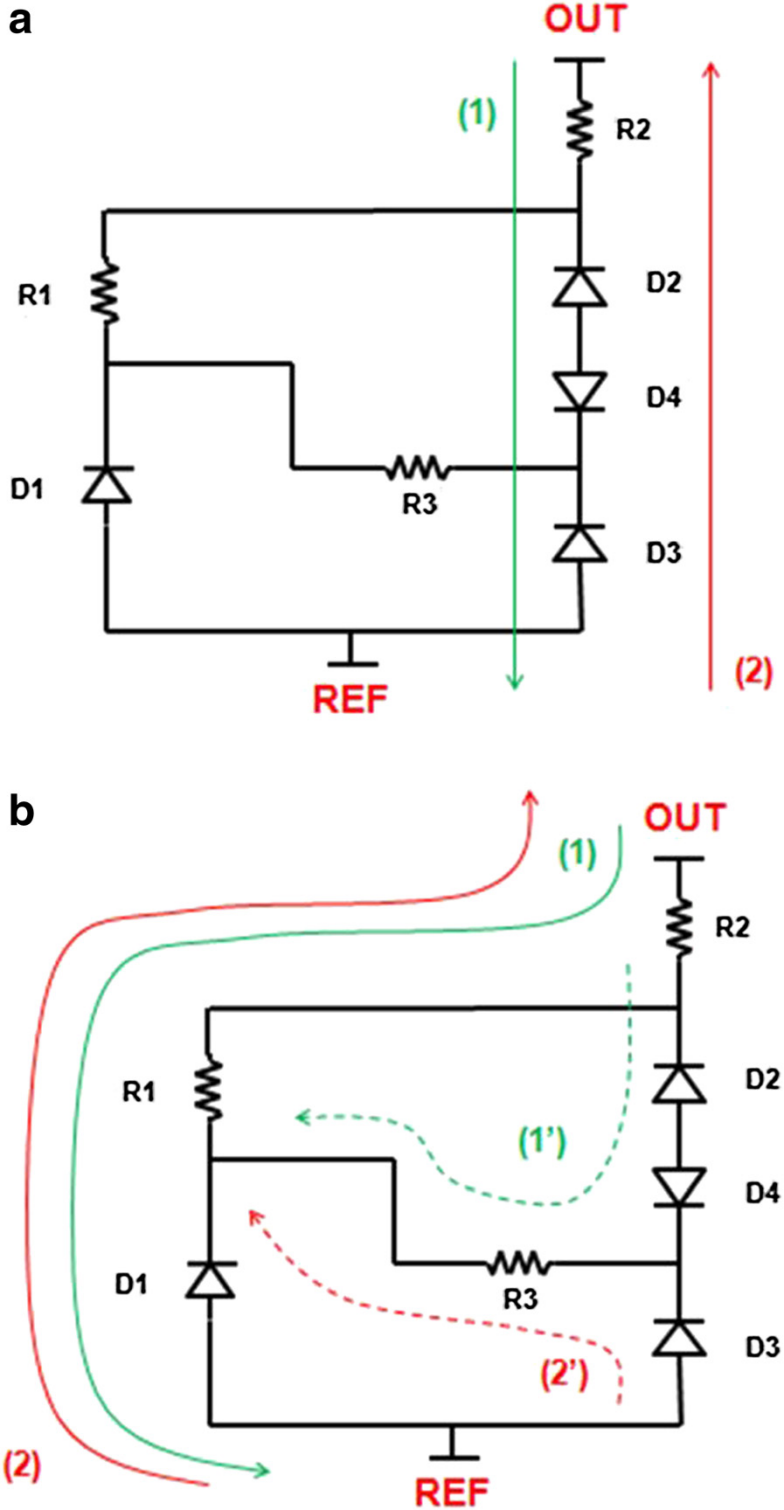
**Hypothetical representations of the current paths inside the experimental structure:** (**a**) if the PS junction termination is not well sized and (**b**) if the limitation comes from the PS layer and its interface with silicon.

From this equivalent schematic, it is now possible to better understand our experimentation results. In fact, we may imagine two main hypotheses as depicted in Figure 11a,b. First, D3 and D4 junctions may be leaky because their PS termination is not well adapted. This case corresponds to Figure 11a. The leakage current flows through R2, D2, D3, and D4. Under forward bias, D4 is conducted and D2 and D3 are blocked. By reversing the bias, all diode behaviors are switched too, so if we consider that D3 and D4 have the same reverse behavior (leaky), the differences observed in Figure 7b,c result only from D2. Higher blocking voltages are reached under forward bias as D2 is off. Second, we may consider that the D3 and D4 behaviors are not altered, then the results of Figure 7b,c should be almost independent of them. This case is presented in Figure 11b. Under forward bias, the main current path (1) should circulate through R2, R1, and D1. Note that R1 may be short-circuited by D2, D4, and R3 (current 1′), but it should be negligible as D2 is off. Under reverse bias, current (2) should circulate through the same elements, but this time, D1 is on, thus explaining the lower blocking voltage observed in Figure 7c. Again, a second current path (2′) is possible through D3 and R3, worsening the reverse bias situation.

Both hypotheses allow us to explain the observed electrical behavior; nevertheless, further investigations will be necessary to clarify the involved physical mechanisms and to adjust the conception of the porous silicon-based AC switch periphery.

## Conclusions

We have discussed the possibility of benefiting from the insulating properties of micro/meso porous silicon etching from p-type silicon on AC switch structures. The first results are promising. Indeed, voltage capabilities of about 500 V have been supported by our simplified porous silicon structure, but many further experimentations will be needed before achieving a reliable AC switch device: localization of the porous silicon layer and fabrication of more realistic samples, definition and characterization of specific patterns to better understand electrical properties of all porous silicon layers embedded in the device, and impact of the AC switch applicative environment knowing that porous silicon properties are highly dependent on the ambient atmosphere (reliability meanings).

## Competing interests

The authors declare that they have no competing interests.

## Authors’ contributions

SM wrote the manuscript, manufactured the experimental samples, and performed the electrical characterizations. DV helped in the realization of the samples. AF and GG participated in the bibliographical study. JB and GG participated in the conception of the study and revised the manuscript. All authors read and approved the current manuscript.
